# Adherence to Antibacterial Therapy and Associated Factors in Lower Respiratory Infections in War-Affected Areas: A Randomized Controlled Trial

**DOI:** 10.3390/antibiotics14100977

**Published:** 2025-09-27

**Authors:** Faiz Ullah Khan, Farman Ullah Khan, Haishaerjiang Wushouer, Luwen Shi, Yu Fang

**Affiliations:** 1 Department of Pharmacy Administration and Clinical Pharmacy, School of Pharmacy, Xi’an Jiaotong University, Xi’an 710061, China; faiz@bjmu.edu.cn (F.U.K.); farmanullah.khan@cust.edu.pk (F.U.K.); 2 Department of Pharmacy Administration and Clinical Pharmacy, School of Pharmaceutical Sciences, Peking University, Beijing 100191, China; kaiser@bjmu.edu.cn (H.W.); shilu@bjmu.edu.cn (L.S.); 3 International Research Centre for Medicinal Administration (IRCMA), Peking University, Beijing 100191, China; 4 Department of Pharmacy Practice, Faculty of Pharmacy, Capital University of Science and Technology, Islamabad 46000, Pakistan

**Keywords:** lower respiratory tract infections, antibiotics, adherence, intervention, awareness

## Abstract

**Background:** Lower respiratory tract infections (LRTIs) are one of the leading causes of mortality. Pharmacist-led interventions can enhance adherence to antibiotics; the present study aims to determine adherence to the antibiotics prescribed for LRTIs and related factors. **Methods:** An individual randomized controlled trial was conducted with 1:1 (intervention = 205, control = 205) participants aged >18 years. The primary outcomes included adherence to therapy at weeks 1 and 2, awareness of antibiotic use, and appropriate discontinuation as prescribed. The secondary outcome measures were the overall clinical outcomes of the therapy and the effectiveness of educational interventions assessed at the final week 7 (overall assessment checked and treatment was not continued). The data were analyzed using different statistical methods, including descriptive statistics for data summarization, and inferential techniques were used. **Results:** Finally, 187 patients remained in the intervention group, and a total (*n* = 18) lost to follow-up. The awareness was significantly increased through pharmacist-led interventions (*n* = 106, 56.7%; *p* = 0.01), along with the factors leading to antibiotic resistance knowledge. Overall, adherence to antibacterial therapy for the LRTIs has improved (*p* ≤ 0.01), and a significant correlation exists between overall MMAS-8 mean scores and other demographic factors; interventions improved [0.441–2.151] with adherence (post-intervention). Higher adherence was found (*p* ≤ 0.05) among the participants in the intervention group compared to the control group and with (OR: 1.050; CI: 0.150–1.024) demographics (education, *p* = 0.05). **Conclusions:** Overall, the intervention group showed better awareness, understanding, and attitudes about antibiotics, and their adherence to antibiotic therapy improved significantly, along with the overall clinical outcomes.

## 1. Introduction

Lower respiratory tract infections (LRTIs) are still among the most prevalent diseases in the world, even though they are primarily preventable causes of death [1]. LRTI is the fifth-leading cause of death and the main contributor to disability-adjusted life years (DALYs) [1,2,3]. Age, poor socioeconomic situation, low birth weight, air pollution, congestion, malnutrition, and insufficient vaccination are all significant risk factors related to LRTI prevalence [4]. The epidemiology of LRTIs has changed significantly among individuals over the age of 70 in the past ten years [5]. Global research studies from diverse territories, including North America, Latin America, Europe, and the Asia-Pacific region, illustrate that the infections caused by CAP (community-acquired pneumonia) were discovered to primarily include resistance in *S. pneumoniae* [6,7]. Controlling the inappropriate use of antibiotics is one strategy to combat the decline in antibiotic effectiveness brought on by resistance [8]. An effort known as antimicrobial stewardship (AMS) seeks to encourage the responsible and safe use of antibiotics without compromising patient quality of life [9].

Educational interventions can improve the prescription, distribution, and/or use of antibiotics, which are frequently used as part of a combination of treatments. Community-based educational programmes increased knowledge and attitudes around the use of antibiotics and reduced the rates of antibiotic prescriptions [10]. Furthermore, the subject matter has not received much attention in Pakistan, particularly when it comes to thorough education initiatives for patients who visit pharmacies to increase adherence to antibiotic treatment and reduce needless medication storage in the home [11,12]. According to recent studies, pharmacy care services can help patients better adhere to their medicines [13,14].

Although wide-ranging experimental studies on antibiotic adherence in LRTI patients remain limited, current data indicate that targeted interventions can be more applicable. The tailored interventions can optimize antibiotic use along with adherence to antibiotics and have demonstrated significant benefits for patients with LRTIs in the experimental group [15]. Multidimensional approaches targeting general practitioners have been shown to improve adherence to first-line antibiotic prescribing suggestions for RTI patients, which led to positive treatment outcomes [16]. Correspondingly, interventions to increase adherence to clinical guidelines have reduced both the volume of antibiotic prescriptions and the inappropriate selection of antibiotics for RTI patients [17]. In geriatric populations, complete reviews have recognized the most promising antibiotic regimens for LRTI management in both hospital and outpatient settings [18]. Recently, specific interventions have determined significant improvements in key quality indicators for CAP management, supporting the effectiveness of multifaceted stewardship plans in improving clinical practice and decreasing resistance [19]. Moreover, pharmacists can assist patients with LRTIs and provide them with guidance on the appropriate use of their prescribed antibiotic treatment [20,21]. Research on pharmacist-led interventions to improve pharmacotherapy has revealed mixed findings, and there is a lack of large, comprehensive studies in community care settings to strengthen evidence-based practice [22]. Medication non-adherence is a common issue in clinical practice, whereas adherence greatly influences treatment clinical outcomes [23].

More than 50% of patients do not take their medicines as prescribed, which could result in the emergence of antibiotic resistance [24]. Based on a study of 63 studies conducted over 30 years, scientists found that adherent patients have a threefold higher likelihood of receiving a favourable result than non-adherent patients [25,26]. In the case of infectious disorders, non-adherence with antibiotics may result in the storage of medicines at home, which encourages self-medication, resulting in a vicious cycle that favours bacterial resistance [27]. Patients must strictly adhere to their prescribed medications for the effective and desired outcomes.

Therefore, the present study aimed to evaluate patients’ adherence to antibiotic therapy for LRTIs, as well as their awareness and the factors associated with adherence.

## 2. Results

**Figure 1:**
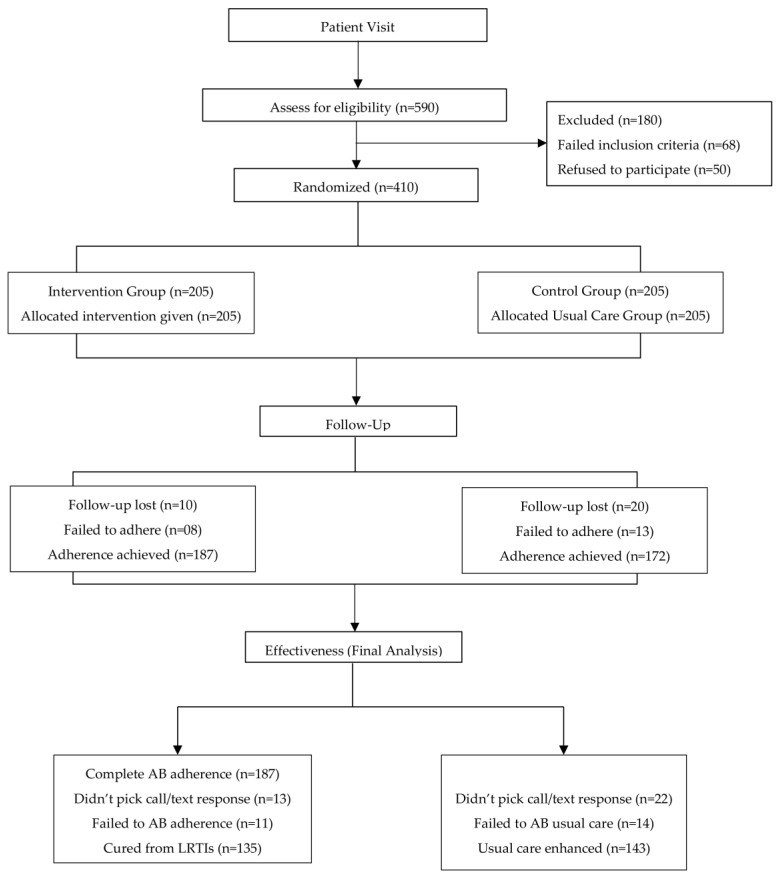
Overall process of this study.

A total of 590 participants were enrolled in this study. Of those, 410 were eligible for randomization post-screening (CP-I: *n* = 100, CP-II: *n* = 110, CP-III: *n* = 102, and CP-IV: *n* = 98), and 205 individuals were assigned to each group, while 180 were excluded. All patients consented to take part in the trial. Patients were assigned to one of the two groups (IG, *n* = 205; CG, *n* = 205) from the two UCs of Khwazakhela Swat. A total lost to follow-up were *n* = 18 and failed to achieve intervention in the IG, and, finally, 187 participants were included (Figure 1).

Overall, the majority of the participants were male in the IG (*n* = 176, 94.1%), with the mean age group of 18–23 (*n* = 59, 31.6%), as well as in the CG (*n* = 187, 91.2%), with the mean age group of 18–23 (*n* = 58, 28.3%). The demographic variables of patients (ARTIs *n* = 98, AECOPD *n* = 45, CAP *n* = 37, COPD *n* = 20) are reported (Table 1).

Most of the participants had a basic understanding of the term “antibiotics.” In both groups, respondents defined the antibiotics as the pills that cure infection, antibiotics in the CG (*n* = 134, 65.6%) vs. IG (*n* = 135, 72.2%; *p* = 0.00). Antibiotics used for the present illness, including penicillin (amoxiclav) in the CG (*n* = 40, 19.5%) vs. (*n* = 44, 21.4%) refill for the same class in the IG (*n* = 1, 0.5%) and polypharmacy, were observed for the penicillin class with other antibiotics in the CG (*n* = 20, 9.5%) vs. IG (*n* = 19, 10.1%) and macrolides (Azithromycin and Clarithromycin) in the CG (*n* = 30, 14.6%) vs. IG (*n* = 18, 9.7%) and commonly prescribed in the present illness of the CG (*n* = 89, 43.5%) vs. IG (*n* = 62, 30.2%) (Supplementary Material Table S1).

### 2.1. Primary Outcomes

The primary outcome was patient adherence to the prescribed antibiotic regimen, and general knowledge of the participants was assessed with different responses. Most of the respondents in the CG believe that coloured mucus can be treated with antibiotics, as CG (*n* = 134, 65.6%) vs. IG with no response (*n* = 43, 23.0%; *p* = 0.01). Not all cases of persistent cough need antibiotic therapy as CG (*n* = 0, 0.0%) vs. IG (*n* = 30, 16.0%). On the other hand, sore throat, persistent cough and cold, flu, along with fever, were mixed with chest infections or LRTIs in many of the participants in the CG (*n* = 140, 68.2%) vs. IG (*n* = 103, 55.1%). Overall, a significant increase in the knowledge was observed of the IG as given in Supplementary Material Table S2. The WHO (PAS-QA) responses were recorded for most of the participants who used antibiotics in the last month: CG (*n* = 40, 19.5%) vs. IG (*n* = 30, 16.0%). In the past 6 months, CG (*n* = 56, 27.3%) versus IG (*n* = 41, 21.9%), and more than 50% of the respondents used antibiotics in the past year: CG (*n* = 109, 53.2%) vs. IG (*n* = 116, 62.0%). On that occasion, participants tend to get antibiotics from a doctor or a nurse first aid clinic in the locality and get advice in the form of marking on the medicine packs: CG (*n* = 184, 89.8%) vs. IG (*n* = 157, 84.0%). The source of information responses is shown in Supplementary Material Figure S1.

Antibiotic resistance can be linked with overuse or regular consumption, with the correct and incorrect answers recorded (Supplementary Material Table S3). Bacterial resistance to a specific antibiotic can spread and transfer from one person to another: CG (*n* = 88, 42.9%) vs. IG (*n* = 113, 60.4%) (Supplementary Material Table S4). The presence of a duty pharmacist at CPs gave brief information on the antibiotics to the participants and dispensed them mainly on prescription. Additionally, Table 2 also presents the median and IQR of both groups in detail.

The BMQ-QA responses are in the LS from “SA to SD” format, and each question has its code. C1 worries about the medicine taken at present: CG with ‘A’ option (*n* = 59, 28.8%) vs. IG (*n* = 163, 79.5%). C3, participants found the present medicine CG with D option (*n* = 74, 36.1%) vs. IG (*n* = 65, 34.1%). The details about the BMQ-QA are given in Table 3. C4, the current medicine disruption with the participant’s life, found no significant association with CG with D option (*n* = 73, 35.6%) vs. the IG (*n* = 70, 37.4%). N5, medication protection against worsening has no significant association with CG, with ‘A’ option (*n* = 143, 69.8%) vs. IG (*n* = 120, 64.2%). C6, CG with ‘A’ option (*n* = 161, 69.8%) vs. IG (*n* = 152, 81.3%). Moreover, Table 3 provides a detailed comparison of the median and IQR for both groups.

### 2.2. Secondary Outcomes

The clinical improvement has been achieved, and overall MMAS-8 scores were improved in the IG than CG, as presented in Table 4, and overall percentages were taken for both groups. Participants miss their medication intentionally, and, in the last weeks, respondents have responded with yes: CG (*n* = 96, 54.4%) vs. IG (*n* = 81, 45.8%). During travels and when leaving home, participants always forget their medications.

**Figure 2:**
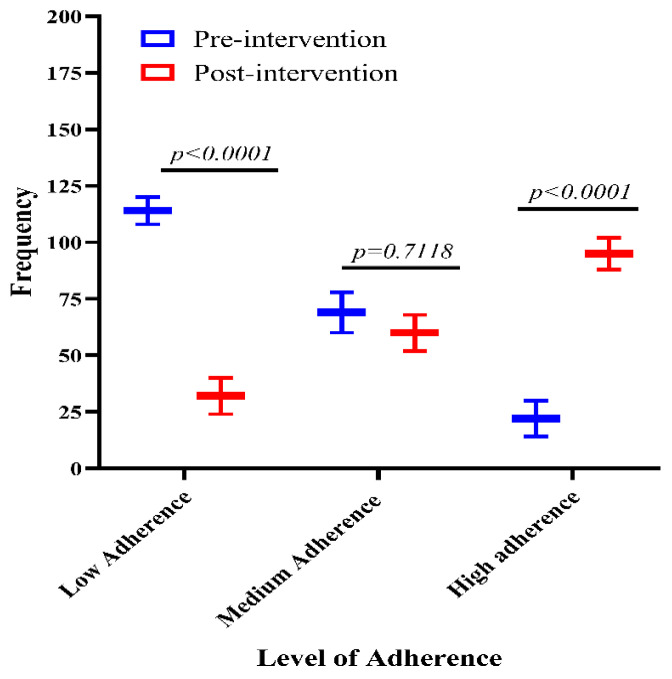
Overall, MMAS-8 measures in pre- and post-interventions.

The overall MMAS-8 was divided into lower (score 1–2), medium (score 2–3), and higher (>6) adherence. The participants had low adherence in the CG (*n* = 125, 61.0%) vs. IG (*n* = 32, 17.1%), medium adherence in the CG (*n* = 66, 32.0%) vs. IG (*n* = 60, 32.1%), and higher adherence was found in the CG (*n* = 14, 6.8%) vs. IG (*n* = 95, 50.8%). The overall results show that the adherence to antibacterial therapy for the LRTIs in the IG group has significantly improved (*p* ≤ 0.01) as compared to the CG (Figure 2).

### 2.3. Comparison of the Demographics

Different sociodemographic variables were checked against the MMAS-8 scores, as education has a significant (*p* < 0.05) association. The overall MMAS-8 mean scores have a significant (*p* ≤ 0.01) association with other demographic variables, CG (6.4 ± 2.1) vs. IG (7.8 ± 2.1). Overall, the demographic factors are linked with higher adherence compared to the medium group and the intermediate education group that followed (*p* = 0.089), with interventions [0.441–2.151]. Upon the final analysis, the multinomial regression model findings are presented in Table 5. The age group of 30–31 is more likely to show high adherence (*p* = 0.080) than other groups, with the odds ratio (OR: 2.040) and confidence interval [CI: 0.637–6.536]. The majority of educational interventional participants get healthier after the prescribed ABs are used correctly (Supplementary Material Table S5).

The financial situation is one of the main factors, and the monthly income is more than or equal to PKR 30000 (*p* = 0.006) to the ABs (OR:4.088) as per education received on prescribed ABs [1.488–11.229]. The analysis of the relationship between past medical history and adherence yielded a non-significant association (*p* > 0.05), indicating that a patient’s health background, whether distant or recent, does not significantly impact adherence to prescribed treatments. A noteworthy observation arises regarding intervention efficacy, suggesting that interventions administered at the onset of the present illness may play a crucial role in reducing non-adherence in the future. This strategic timing of interventions holds promise for enhancing patient adherence, potentially contributing to improved overall treatment outcomes.

The outcomes met the objectives of the RCT, and adherence was measured as higher in the IG as compared to the usual care and other categories. Gender is less likely to be a factor in adherence with the prescribed medications and is not influenced or changed (*p* > 0.05). The education secondary group (OR: 1.050) shows more adherence (*p* = 0.056) to the prescribed treatment with ABs as compared to other classifications [CI: 0.150–1.024]. The conclusive results of the randomized controlled trial (RCT) demonstrated the achievement of this study’s objectives. Notably, adherence rates within the IG surpassed those observed in the usual care group and other relevant categories. Remarkably, gender did not emerge as a significant factor influencing adherence to prescribed medications, as indicated by a non-significant *p*-value (*p* > 0.05). Upon closer examination of demographic variables, it was found that individuals in the secondary education group exhibited a slightly higher likelihood of adherence to the prescribed antibiotic treatment (OR: 1.050). While the *p*-value (0.056) suggests a trend toward significance, it falls just short of the conventional threshold. The confidence interval (CI: 0.150–1.024) further provides a range within which the true odds ratio is likely to exist. This implies that, although not statistically significant, there is a potential trend indicating greater adherence in the secondary education group compared to other classifications. The results based on the Mann–Whitney U test, comparing responses across demographic groups (Supplementary Material Tables S6.1 and S6.2) in detail.

In essence, this study’s outcomes underscore the efficacy of the intervention in promoting adherence, with noteworthy insights into demographic factors such as education level. While gender did not exert a discernible impact, the nuanced analysis of subgroups sheds light on potential variations in treatment adherence, particularly within the secondary education category. These findings contribute valuable information to the broader understanding of factors influencing adherence to prescribed medications.

## 3. Discussion

This is the first of its kind study examining antibiotic adherence in the LRTI patients from Pakistan post-conflict areas. The awareness and adherence have been improved, and the study group has become more compliant with the advised antibacterial therapy.

The overuse and non-adherence to antibiotic therapy have been highlighted by cross-sectional studies, point prevalence surveys, and significant work on these dimensions, which demonstrate the serious concern surrounding the irrational use of antibiotics in infectious diseases [28]. U. Taj and the authors raised awareness of the challenges that Pakistanis face when attempting to take prescription antibiotics, but our study has tried to reduce these challenges by increasing adherence [29]. The sharing of antibiotics among friends and family members is still common and comparable to that of other lower-income nations. According to research from the Philippines, there are misconceptions about antibiotic sharing with others and its relationship to demographics. A Saudi study by AAH Alenazi et al. reported that the term “resistant bacteria” is mostly known by coworkers and households through social media platforms [30]. Similar to results reported by Scott A. McEwen and his colleagues, our study has improved such factors, particularly the households with less familiarity with antibiotic resistance [31,32]. Participants’ health is dependent on their existing medications, and nearly the same finding was reported by Rachael J. Thorneloe and his colleagues [23].

The non-adherence includes not following prescriptions completely, skipping doses, using the wrong dosage intervals, and stopping medications too soon. There have been reports of non-adherence in 20–80% of cases, on average in the middle at 50–60% [33]. In Pakistan, inappropriate use of antibiotics is linked to several problems, including poor health literacy, low educational attainment, the expense of private practitioners, patient overcrowding, busy schedules, and an unmanaged medicine supply [34,35]. While Kuntz et al. highlighted various intentional and unintentional non-adherent behaviours requiring further treatment, our study found that most participants in the IG adhered to initial therapy and remembered to take their first dose [36]. Lornia and Maria reported that an educational intervention that contained information on antibiotic use and resistance significantly improved patient adherence, reduced antibiotic wastage, and highlighted that stronger general-overuse beliefs were associated with increased non-adherence [37]. The majority of patients in the current study continued to take their antibiotics as directed and did not stop taking them, differing from Khan and his colleagues’ earlier findings that most participants had stopped taking them after feeling better [37]. Participants also frequently forget their medications when travelling or leaving home, while, in the post IG, such factors are found to be controlled. All the efforts suggested that pharmacist-led intervention improved adherence with recommended antibiotics in the given population.

The present study has limitations too; first, the findings cannot be generalized to the entire country, as this study was conducted in post-conflict areas where adherence rates may be affected and a small proportion of female participation. Second, the study follow-up was ensured through reminder messages; however, contacting patients on time remained challenging. Due to the study protocols, the leak of information might be possible; patients may share intervention details with others, potentially affecting the CG participants nearby locality.

These findings have important implications for future scale-up in similar LMIC settings, where targeted educational interventions and strengthened healthcare infrastructure could significantly improve antibiotic adherence and reduce misuse.

## 4. Materials and Methods

### 4.1. Study Design

The present study is a single-blinded, two-arm, individual trial carried out to examine the effectiveness of pharmacist-led educational interventions for patients approaching community pharmacies (CPs) with prescriptions containing antibiotics for LRTIs.

### 4.2. Study Setting, Population, and Sample Size

This study was conducted in Swat, Khyber Pakhtunkhwa, Pakistan, which has over 2 million inhabitants, around 86% of whom live in rural areas. Infectious diseases, especially LRTIs, are still prevalent in Swat. The study area was influenced by military operations (2007–2009) and declared secure in 2009. The post-conflict challenges make it a key area for studying antibiotic adherence and awareness for the LRTI population. This study was conducted in two union councils (UCs) in the Swat district (Khwazakhela and Janu). In Pakistan’s new local government rules of administration, UC and neighbourhood councils (NHC) are the fundamental entities, with 2000–10,000 people per UC. A total of 200 participants were recruited in each group.

A sample size of the responses was achieved with a 95% confidence interval and a margin of error (5%), with the aim of a 1:1 allocation ratio in the intervention and control groups. A sample size of 400 (200 in each group) was assigned with a proposed 20% attrition rate for the dropout participants as per the published protocols. Sensitivity analysis was conducted for the potential impact of attrition on study and validity; we conducted both intention-to-treat (ITT) and per-protocol (PP) analyses. In the ITT analysis, all randomized participants were included, with missing data addressed using the last observation carried forward (LOCF) method. The PP analysis involved only those participants who completed this study according to protocol.

### 4.3. Data Teams’ Eligibility Criteria and Training

The DCT (data collection team) was most likely made up of healthcare staff (pharmacists, vaccinators, and female health workers). Pharmacists who received special training were assigned CPs (5 = CG: control group, 5 = IG: intervention group), where participants were recruited with complete permission. Before the trial began, effective communication skills training was provided, as well as pseudo-patient elements of training to measure counselling abilities at CPs.

### 4.4. Interventions

Based on available scientific evidence, criteria for antibiotic adherence were devised, including a short telephone-based educational intervention approach that was utilized. The EATSA (effectiveness of adherence to the specific antibiotics) pre-designed protocol was published earlier than the main study [38]. First, IG participants receive written information on the usage of ABs and adherence to LRTI antibiotic therapy, as well as advice about antibiotic storage at home, from a competent pharmacist and DCT. A brochure with information based on “*Get Smart: Know; When Antibiotics Work*” was produced. With the assistance of specialists, the pamphlets were adapted from WHO (World Health Organization)/CDC (Centers for Disease Control and Prevention) public awareness campaigns and translated into national (Urdu/Pushto) and local languages for better comprehension, as shown in Supplementary Material Figure S2. The bottom portions of the booklet included information on the necessity of antibiotic adherence and forgetting, proper disposal, storage counselling, and the importance of not sharing your antibiotics with others.

Secondly, trained pharmacists made visits to the consenting participant households and completed educational training on adherence and storage of antibiotics. A total of 3 sessions were conducted for the households. The WHO guidelines “*How to Investigate the Use of Medicines by Consumers*” were used for the consumption of medicines and aimed to enhance the rational use of antibiotics. The phone calls occurred early in the first week and then continued into the second week. The therapeutic success was determined by the ratio of those who followed through with their commitments to the recommended therapy. Then, on the third, fourth, fifth, and sixth calls, the participants followed up with a brief instructional technique to improve their understanding of how to utilize antibiotics properly.

### 4.5. Usual Care Improvement

Individually, both the IG and CG participants continued to receive pre-arranged regular phone calls, but the CG only obtained daily guidance for their antibiotics, with the treatment being classified as enhanced care. There were no placebos used in primary care for therapy, and the focus was on sticking to the recommended medication.

### 4.6. Inclusion Criteria

If patients with LRTIs obtained complete consent, they were examined for inclusion criteria at the CPs. For adult patients, regardless of gender, one of the study’s qualifying criteria is that participants must have resided in the study area for at least one year and be able to communicate in Urdu/Pushto or English (Supplementary Material Table S7). The participants in the IG arm had no other comorbidities and had not engaged in or been valuable members of any other study/trial-related RTIs or antibiotics in the past 4 months. Participants who were unable to properly answer the questions due to sight/vision, hearing, or cognitive difficulties, as well as those who did not meet the inclusion criteria, were disqualified and eliminated. A random digit number was generated through computer-based allocation. For each recruited participant, a 1:1 sample was utilized to assign them to the CG and IG. The status of allotment was conveyed to DCT administrators, who subsequently contacted individual pharmacists as soon as DCTs had obtained permission from the participants to participate in the trial. Before being randomly assigned to either the CG or IG arms, it was necessary to get informed consent as well as eligibility. The randomization point was kept a secret from research assistants, participants, and trial representatives (pharmacists).

### 4.7. Data Collection

Initially, qualified pharmacists at CPs collected information from patient medical records (medical notes checked for the diagnosis on prescription), and the results were kept secret from the pharmacist team. Each IG participant received a comprehensive packet of instructional written content. Oral counselling was offered in the region specific to the patients at CPs following textual counselling. The patient’s data was safely transferred to pre-determined data-collecting forms. The valid mobile phone numbers of patients were obtained and saved for future contact throughout the follow-up phase. In both UCs, trial DCTs communicated with participants using cell phones. Patients were requested to show their prescription, including antibiotics, after one week, and the initial therapeutic outcomes were assessed at the beginning of week 1 (initial effect of the antibiotics). After week 1, the second week involved discontinuation (as prescribed) and an enhancement in the knowledge, attitude, and practices initially assessed. Then, three follow-ups were completed: W3, W4, and W5 to assess the overall pharmacist-led interventions and their effectiveness, monitor sustained changes, and detect any relapse or inappropriate re-use of antibiotics. The antibiotics were not continued for long and stopped as prescribed (minimum 5 days to maximum 10 days), and information was given promptly to IG. Data gathering was updated and trustworthy to preserve data quality and dependability. All data were gathered once a week by the principal investigator (PI). We advised patients to read the SMS text messages delivered by our research team after a one-on-one appointment. Four sections of SMS text messages were included: awareness about LRTIs, understanding of antibiotics, modification of daily routine, and improvement of medication adherence. Week 7 marked the final assessment phase, during which trial participants were evaluated for overall outcomes (clinical and awareness-related). Moreover, patients in the intervention arm discontinued antibiotics as directed (in 5–10 days), and none continued antibiotics until week 7. The trial aligned with pre-defined protocols and SPIRIT (Standard Protocol Items: Recommendations for Interventional Trials) guidelines as detailed in the Supplementary Material Table S8.1.

### 4.8. Blinding

Due to its instructional character, blinding was difficult; however, some steps have been taken. Supplementary Material Table S8.2 provides comprehensive information about blinding. To minimize bias, both trial participants and outcome assessors were blinded to the intervention allocation. Blinding was maintained by identical packaging and labelling of both study arms. Specifically, the individuals responsible for collecting and analyzing data related to adherence were not included in delivering the intervention and remained unaware of participant allocation positions.

### 4.9. Outcomes Measures

The primary outcome was patient adherence to the prescribed antibiotic regimen, defined as taking ≥80% of the prescribed doses based on pill count and the enhancement of the knowledge, attitude, and practices of the patients. The secondary outcomes included clinical improvement and indicators like infection-related symptoms as per the adherence score with the outcomes of the therapy.

#### 4.9.1. Primary Measures

The primary outcomes are more likely to be evaluated at time point two (week 1: initial course of antibiotics completion and week 2: initial adherence with awareness), whether the patient experienced any unpleasant effect or adherence to the prescribed therapy after the interventions was assessed, along with related factors. The BMQ (beliefs about medication questionnaire) was used to measure participant attitudes about antibiotics and was purely for the aim of evaluating participant attitudes regarding antibiotics. The primary outcome was to measure the level of the individual’s (LRTI) understanding of ABs with BMQ. The participants were allowed to try out the BMQ. Finally, an 18-item QA with a 4-factor structure that remained consistent across disease groups was developed. The BMQ-QA was validated in a variety of patient groups. The primary outcome of both PAS-QA (public awareness survey questionnaire) and BMQ would be to determine the level of antibiotic knowledge and understanding among LRTI patients. The internal consistency was checked through the Cronbach alpha test (>7 each), and the questionnaire’s cultural adaptability was ensured through the modified version of the BMQ and MMAS-8 (Morisky Medication Adherence Scale-8). Later, the questionnaires were translated into the local language (Urdu) as modified previously.

#### 4.9.2. Secondary Measures

The effectiveness of the educational intervention on adherence to the recommended LRTI treatment was assessed based on overall clinical outcomes at week 7, even though the antibiotic treatment had already ended at week 1 or 2. Overall, adherence was taken as the outcome, with other factors to determine whether cured or not, and adhering or not to the recommended therapy. The first 07 questions on the MMAS-8 adherence scale need a yes or no (dichotomous) response, while the latter five are on a Likert scale. The “Pill Count” is a simple novel technique and method to assess patient medication adherence, particularly in antibiotics. The master assessments of the LRTI acceptance plan followed, as demonstrated by the terms “non-adherent” or “adherent” appearing on previously circulated tests. The current research group concluded that a patient who completes the first would be projected as one “disciple” with MMAS-8, 6, and an 80.0% rate of pill count.

### 4.10. Data Management

Data were managed per established procedures. Every day, the data was stored on a hardcopy evaluation study booklet with a participant’s name and codes assigned to each data file by the PI field office. The PI double-checked the data, addressed any issues accordingly, and a third person corrected any inconsistencies. The intervention’s confidentiality is known only to pharmacists, and all data forms are maintained in locked file cabinets. Daily, the trial team monitored this study’s progress.

### 4.11. Data Analysis

IBM-SPSS-Statistics (version 24.0) was used for the analysis of the data. Both descriptive (summarization) and inferential statistical (effect and associations) tests were used. The WHO-PAS and BMQ questionnaires were scored on a 5-point Likert Scale, with total and domain-specific scores computed and summarized as median with interquartile range (IQR); group comparisons were carried out by Mann–Whitney U statistics, while mean (SD) values were additionally calculated to descriptively summarize group-level differentiations (Supplementary Material Table S9). At each visit, the mean differences (MDs) between the two arms, with a 95% CI, for every visit from the IG were combined in a generalized mixed model. All analyses were established in full according to statistical plans assigned before the trial could be unmasked.

### 4.12. Ethical Approval, Consent, Registration, and Trial Status

The ethical approval was provided by the Xi’an Jiaotong University ethical committee with reference No: 2020–13541-XJTU and the project for further process and MMAS-8 questionnaire permission licence. The current study protocols were reviewed by a scientific expert in SGTHs (Saidu Group of Teaching Hospitals), Swat, and PIMS (Pakistan Institute of Medical Sciences). The trial protocol can be found on the website ChiCTR (http://www.chictr.org.cn/) with the reference number ChiCTR2000040453 and published version [38]. Informed consent was obtained before the data collection on an individual basis, and the challenges in the ethical consideration were discussed with the help of the DCT and pharmacists. CONSORT (Consolidated Standards of Reporting Trials) guidelines were used in this study, as detailed information was given in a separate checklist in Supplementary Material File S1.

## 5. Conclusions

Overall, the intervention group showed improved awareness, understanding, and attitudes toward antibiotics, with significantly better adherence to antibiotic therapy. The primary and secondary outcomes were achieved, with high adherence observed among LRTI patients to their prescribed treatment.

## Figures and Tables

**Table 1:** Demographic variables of the patients.

Characteristics	Control *n* (%)	Intervention *n* (%)
Gender		
Male	187 (91.2)	176 (94.1)
Female	18 (8.8)	11 (5.9)
Age (years)		
18–23	58 (28.4)	59 (31.6)
24–29	50 (24.4)	47 (25.1)
30–35	37 (18.0)	41 (21.9)
36–41	37 (18.0)	29 (15.5)
>41	23 (11.2)	11 (5.9)
Education		
Illiterate	33 (16.1)	26 (13.9)
Primary/Secondary	84 (41.0)	56 (29.9)
Intermediate	70 (34.1)	74 (39.6)
Bachelor’s or higher	18 (8.8)	31 (16.6)
Monthly income (PKR *)		
>10,000–<20,000	70 (34.1)	61 (32.6)
>20,000–<300,000	68 (33.2)	69 (36.9)
>30,000–<40,000	34 (16.6)	39 (20.9)
>40,000–<50,000	2 (1.0)	4 (2.1)
>50,000	31 (15.1)	14 (7.5)
Past medical history		
Yes	60 (29.3)	54 (28.9)
No	145 (70.7)	133 (71.1)

* PKR = Pakistani rupees.

**Table 2:** WHO questionnaire responses of the participants.

WHO-QA	Response	Control	Median (IQR)	Intervention	Median (IQR)
Animals raised for food should receive fewer antibiotics from farmers.	Strongly agree	40 (19.5)	4 (1)	113 (60.4)	4 (0)
	Agree	92 (44.9)		16 (8.6)	
	Neutral	43 (21.0)		21 (11.2)	
	Disagree	13 (6.3)		17 (9.1)	
	Strongly disagree	17 (8.3)		20 (10.7)	
People shouldn’t store ABs and use them for different conditions in the future.	Strongly agree	10 (4.8)	4 (3)	120 (64.2)	4 (1)
	Agree	110 (53.7)		0 (0.0)	
	Neutral	68 (33.2)		45 (24.1)	
	Disagree	15 (7.3)		19 (10.2)	
	Strongly disagree	2 (1.0)		3 (1.5)	
Caregivers must check the status of each of their children’s immunizations.	Strongly agree	16 (7.8)	4 (1)	132 (70.6)	4 (2)
	Agree	121 (59.0)		0 (0.0)	
	Neutral	55 (26.8)		43 (23.0)	
	Disagree	3 (1.5)		3 (1.6)	
	Strongly disagree	10 (4.9)		9 (4.8)	
	Strongly disagree	0 (0.0)		0 (0.0)	
Doctors should only issue antibiotic prescriptions when necessary.	Strongly agree	7 (3.4)	4 (0)	121 (64.7)	4 (1)
	Agree	109 (53.2)		0 (0.0)	
	Neutral	63 (30.7)		31 (16.6)	
	Disagree	18 (8.8)		23 (12.3)	
	Strongly disagree	8 (3.9)		12 (6.4)	
If I take my antibiotics as prescribed, I won’t be in danger of developing an antibiotic-resistant infection.	Strongly agree	0 (0.0)	4 (0)	152 (81.3)	4 (0)
	Agree	0 (0.0)		33 (17.6)	
	Neutral	25 (12.2)		2 (1.1)	
	Disagree	155 (75.6)		0 (0.0)	
	Strongly disagree	25 (12.2)		0 (0.0)	
I’m concerned about how antibiotic resistance having will affect my family and my health.	Strongly agree	32 (15.6)	4 (0)	143 (76.5)	4 (2)
	Agree	136 (66.3)		12 (6.4)	
	Neutral	37 (18.1)		32 (17.1)	
	Disagree	0 (0.0)		0 (0.0)	
	Strongly disagree	0 (0.0)		0 (0.0)	
One of the largest issues facing the globe is antibiotic resistance.	Strongly agree	21 (10.3)	4 (0)	118 (63.1)	4 (1)
	Agree	120 (58.5)		14 (7.5)	
	Neutral	54 (26.3)		44 (23.5)	
	Disagree	10 (4.9)		11 (5.9)	
	Strongly disagree	0 (0.0)		0 (0.0)	
Antibiotic resistance will be resolved by medical professionals before it gets out of hand.	Strongly agree	15 (7.3)	4 (1)	95 (50.8)	4 (3)
	Agree	93 (45.4)		0 (0.0)	
	Neutral	49 (23.9)		39 (20.9)	
	Disagree	1 (0.5)		52 (27.8)	
	Strongly disagree	47 (22.9)		1 (0.5)	

**Table 3:** Belief about medication (BMQ) responses of the participants.

	BMQ-QA	Response	Control	Median (IQR)	Intervention	Median (IQR)
N1	My health, at present, depends on this medicine.	Strongly agree	38 (18.5)	3 (2)	43 (23.0)	2 (1)
		Agree	59 (28.8)		93 (49.7)	
		Neutral	35 (17.1)		20 (10.7)	
		Disagree	73 (35.6)		31 (16.6)	
		Strongly disagree	0 (0.0)		0 (0.0)	
C1	Having to take this medicine worries me.	Strongly agree	0 (0.0)	2 (0)	0 (0.0)	2 (1)
		Agree	163 (79.5)		127 (67.9)	
		Neutral	35 (17.1)		37 (19.8)	
		Disagree	7 (3.4)		23 (12.3)	
		Strongly disagree	0 (0.0)		0 (0.0)	
N2	My life would be impossible without this medicine.	Strongly agree	0 (0.0)	3 (2)	0 (0.0)	4 (1)
		Agree	70 (79.5)		62 (33.2)	
		Neutral	5 (17.1)		7 (3.7)	
		Disagree	76 (3.4)		68 (36.4)	
		Strongly disagree	54 (0.0)		50 (26.7)	
C2	I sometimes worry about the long-term effects of this medicine.	Strongly agree	0 (0.0)	2 (1)	0 (0.0)	2 (1)
		Agree	139 (67.8)		137 (73.3)	
		Neutral	28 (13.7)		20 (10.7)	
		Disagree	24 (11.7)		20 (10.7)	
		Strongly disagree	14 (6.8)		10 (5.3)	
N3	Without this medicine, I would be very ill.	Strongly agree	24 (11.4)	2 (2)	0 (0.0)	2 (0)
		Agree	133 (64.8)		148 (79.1)	
		Neutral	24 (11.7)		19 (10.2)	
		Disagree	17 (8.7)		16 (8.6)	
		Strongly disagree	7 (3.4)		4 (2.1)	
C3	This medicine is a mystery to me.	Strongly agree	9 (4.3)	4 (3)	0 (0.0)	4 (2)
		Agree	69 (33.7)		61 (32.5)	
		Neutral	1 (0.5)		13 (7.0)	
		Disagree	74 (36.1)		65 (34.8)	
		Strongly disagree	52 (25.4)		48 (25.7)	
N4	My health in the future will depend on this medicine.	Strongly agree	14 (6.8)	2 (0)	0 (0.0)	2 (1)
		Agree	157 (76.6)		122 (65.2)	
		Neutral	27 (13.2)		29 (15.5)	
		Disagree	7 (3.4)		5 (2.7)	
		Strongly disagree	0 (0.0)		31 (16.6)	
C4	This medicine disrupts my life.	Strongly agree	10 (4.9)	4 (2)	0 (0.0)	4 (1)
		Agree	67 (32.7)		65 (34.8)	
		Neutral	2 (1.0)		0 (0.0)	
		Disagree	73 (35.6)		70 (37.4)	
		Strongly disagree	53 (25.8)		52 (27.8)	
C5	I sometimes worry about becoming too dependent on this medicine.	Strongly agree	14 (6.8)	2 (0)	19 (10.2)	2 (1)
		Agree	160 (78.0)		143 (76.5)	
		Neutral	18 (8.8)		15 (8.0)	
		Disagree	12 (5.9)		9 (4.8)	
		Strongly disagree	1 (0.5)		1 (0.5)	
N5	This medicine protects me from becoming worse.	Strongly agree	14 (6.8)	2 (0)	37 (19.8)	2 (1)
		Agree	143 (69.8)		120 (64.2)	
		Neutral	24 (11.7)		12 (6.4)	
		Disagree	17 (8.3)		12 (6.4)	
		Strongly disagree	7 (3.4)		6 (3.2)	
C6	This medicine gives me unpleasant side effects.	Strongly agree	8 (6.8)	2 (2)	0 (0.0)	2 (0)
		Agree	161 (71.9)		152 (81.3)	
		Neutral	29 (12.7)		29 (15.5)	
		Disagree	7 (8.6)		6 (3.2)	
		Strongly disagree	0 (0.0)		0 (0.0)	

**Table 4:** Scores based on MMAS-8.

Questions	Response	Control	Intervention
		*n* (%)	*n* (%)
Do you sometimes forget to take your medication?	Yes	0 (0.0)	0 (0.0)
	No	205 (52.3)	187 (47.7)
People sometimes miss taking their medicines for reasons other than forgetting. Thinking over the past 2 weeks, were there any days when you did not take your medicine?	Yes	96 (54.2)	81 (45.8)
	No	109 (50.7)	106 (49.3)
Have you ever cut back or stopped taking your medication without telling your doctor, because you felt worse when you took it?	Yes	10 (100.0)	0 (0.0)
	No	195 (51.0)	187 (49.0)
When you travel or leave home, do you sometimes forget to bring along your medication?	Yes	9 (100.0)	0 (0.0)
	No	196 (51.2)	187 (48.8)
Did you take your medicine yesterday?	Yes	17 (34.7)	32 (65.3)
	No	173 (50.4)	170 (49.6)
When you feel like your symptoms are under control, do you sometimes stop taking your medicine?	Yes	91 (52.9)	81 (47.1)
	No	114 (51.8)	106 (48.2)
Taking medication every day is a real inconvenience for some people. Do you ever feel hassled about sticking to your treatment plan?	Yes	90 (52.6)	81 (47.4)
	No	115 (52.0)	106 (48.0)
How often do you have difficulty remembering to take all your medications? (Please circle the correct number)	Never/rarely	125 (85.0)	22 (15.0)
	Once in a while	10 (90.9)	1 (9.1)
	Sometimes	36 (62.1)	22 (37.9)
	Usually	30 (50.8)	29 (49.2)
	All the time	104 (88.9)	13 (11.1)

**Table 5:** Regression analysis for the relationship of adherence with demographic variables.

Groups	Determinants	Odd Ratios	95% CI [Lower–Upper]	*p*-Value **
Adherence Group (higher adherence)	Gender			
	Male	1.767	[0.604–5.172]	0.299
	Female	Reference *	Reference *	
	Age group (years)			
	18–23	1.640	[0.531–5.066]	0.049
	24–29	1.157	[0.369–3.630]	0.390
	30–35	2.040	[0.637–6.536]	0.080
	36–41	1.641	[0.500–5.388]	0.230
	>41	Reference *	Reference *	Reference *
	Education group			
	Illiterate	1.579	[0.301–2.634]	0.833
	Primary/Secondary	1.050	[0.150–1.024]	0.056
	Intermediate	1.316	[0.313–1.829]	0.535
	Bachelor’s or higher	Reference *	Reference *	Reference *
	Monthly income (PKR)			
	>10,000–<20,000	1.899	[0.714–5.048]	0.199
	>20,000–<300,000	4.088	[1.488–11.229]	0.006
	>30,000–<40,000	1.423	[0.501–4.043]	0.508
	>40,000–<50,000	3.402	[0.367–31.496]	0.281
	>50,000	Reference *	Reference *	Reference *
	Medical history			
	Yes	1.226	[0.674–2.232]	0.505
	No	Reference *	Reference *	Reference *
Adherence Group (medium adherence)	Gender			
	Male	2.340	[0.681–8.043]	0.177
	Female	Reference *	Reference *	
	Age group (years)			
	18–23	1.295	[0.551–3.040]	0.919
	24–29	0.769	[0.321–1.845]	0.279
	30–35	1.111	[0.444–2.781]	0.701
	36–41	0.438	[0.158–1.209]	0.112
	>41	Reference *	Reference *	Reference *
	Education group			
	Illiterate	0.890	[0.627–3.973]	0.240
	Primary/Secondary	0.391	[0.432–2.091]	0.332
	Intermediate	0.756	[0.441–2.151]	0.089
	Bachelor’s/higher	Reference *	Reference *	Reference *
	Monthly income (PKR)			
	>10,000–<20,000	1.482	[0.560–3.924]	0.429
	>20,000–<300,000	1.919	[0.692–5.319]	0.210
	>30,000–<40,000	1.391	[0.507–3.818]	0.521
	>40,000–<50,000	2.858	[0.321–5.459]	0.347
	>50,000	Reference *	Reference *	Reference *
	Medical history			
	Yes	1.003	[0.553–1.821]	0.992
	No	Reference *	Reference *	Reference *

* The reference category is as follows: Low adherence: ** Multinomial Regression Analysis: PKR = Pakistani rupees.

## Data Availability

The data can be provided upon reasonable request to the corresponding author.

## Supplementary Materials

The following supporting information can be downloaded at: https://www.mdpi.com/article/10.3390/antibiotics14100977/s1, Figure S1: Different sources of information; Figure S2: Brochure for the participants based on educational intervention; Table S1: Basic knowledge responses towards RTIs; Table S2: The general knowledge of the participants; Table S3: Response towards WHO-PAS-QA; Table S4: Trial participants responses on ABR; Table S5: Comparison of MMAS-8 scores with participant demographics; Table S6.1 & S6.2: The WHO-PAS Likert Scale and BMQ responses and Demographics association; Table S7: Details of a text message description of the contents; Table S8.1 & S8.2: SPIRIT and Blinding; Table S9: Mean differences and overall scores; File S1: CONSORT Checklis.
