# Correction: Khan et al. Adherence to Antibacterial Therapy and Associated Factors in Lower Respiratory Infections in War-Affected Areas: A Randomized Controlled Trial. *Antibiotics* 2025, *14*, 977

**DOI:** 10.3390/antibiotics14121272

**Published:** 2025-12-16

**Authors:** Faiz Ullah Khan, Farman Ullah Khan, Haishaerjiang Wushouer, Luwen Shi, Yu Fang

**Affiliations:** 1 Department of Pharmacy Administration and Clinical Pharmacy, School of Pharmacy, Xi’an Jiaotong University, Xi’an 710061, China; faiz@bjmu.edu.cn (F.U.K.); farmanullah.khan@cust.edu.pk (F.U.K.); 2 Department of Pharmacy Administration and Clinical Pharmacy, School of Pharmaceutical Sciences, Peking University, Beijing 100191, China; kaiser@bjmu.edu.cn (H.W.); shilu@bjmu.edu.cn (L.S.); 3 International Research Centre for Medicinal Administration (IRCMA), Peking University, Beijing 100191, China; 4 Department of Pharmacy Practice, Faculty of Pharmacy, Capital University of Science and Technology, Islamabad 46000, Pakistan

## Text Correction

There was an error in the original publication [1]. The corrected paragraphs are provided below:

1. Section 2.2:

“The overall MMAS-8 scores were categorized as low (<6), medium (6 to <8), and high (8) adherence.”

2. Section 4.9.2:

“In accordance with the MMAS-8 scoring protocol, total scores range from 0 to 8, categorized as low (<6), medium (6 to <8), and high adherence (8), as follows in the current study [39].”

3. Section 4.12:

“Professor Donald E. Morisky, who developed the MMAS-8 (U.S. Copyright Registration TX0008632533), permitted us to use it in this study (Supplementary Material File S2).”

4. Section Supplementary Material:

“File S1: CONSORT Checklist. File S2: Morisky Widget Retroactive & Corrective License.”

## Reference Correction

There is a new citation, number 39.

“39.  Moon, S.J.; Lee, W.Y.; Hwang, J.S.; Hong, Y.P.; Morisky, D.E. Accuracy of a screening tool for medication adherence: A systematic review and meta-analysis of the Morisky Medication Adherence Scale-8. *PLoS ONE* **2017**, *12*, e0187139. https://doi.org/10.1371/journal.pone.0187139.”

The authors state that the scientific conclusions are unaffected. This correction was approved by the Academic Editor. The original publication has also been updated.
