# Supplementary material for: Adherence to Antibacterial Therapy and Associated Factors in Lower Respiratory Infections in War-Affected Areas: A Randomized Controlled Trial
Source: Antibiotics (Basel). 2025 Sep 27;14(10):977. doi: 10.3390/antibiotics14100977 (PMC12561823; doi:10.3390/antibiotics14100977)
Supplement: Supplementary file 1 [file antibiotics-14-00977-s001.zip › 1.Supplementary Table-S1 Basic knowledge responses towards respiratory tract infections.pdf]

Supplementary -Table S1: Basic knowledge responses towards respiratory tract infections

Basic knowledge	Control *n* (%)	Intervention *n* (%)	*P*-value*
**Do you know about RTIs or LRTs?			0.01
Yes	70 (34.1)	103 (55.1)	
No	83 (40.5)	50 (26.7)	
Do not know	52 (25.4)	34 (18.2)	
Do you ever visit the physician for RTIs or LRTIs?			0.05
Yes	102 (50.2)	109 (58.3)	
No	103 (49.8)	78 (41.7)	
Do not know	0 (0.0)	0 (0.0)	
Do you hear/know the term “antibiotics”?			0.00
Yes	134 (65.4)	135 (72.2)	
No	41 (20.0)	29 (15.5)	
Have you ever used antibiotics before?			0.002
Yes	143 (69.8)	166 (88.8)	
No	38 (18.5)	06 (3.2)	
Do not know	24 (11.7)	15 (8.0)	
Have you ever used antibiotics for flu and clod?			0.05
Yes	155 (75.6)	155 (82.8)	
No	27 (13.2)	18 (9.6)	
Do not know	23 (11.2)	14 (7.5)	
Do you get antibiotics for RTIs only on prescription?			0.001
Yes	77 (37.6)	80 (42.7)	
No	111 (54.1)	107 (57.2)	

Table S1 (Continued)

Antibiotics: control-intervention (n=205 – n=187)	For present illness	Refill	Polypharmacy
Penicillin (Amoxiclav and Co-amoxiclav)	40 (19.5) – 44 (21.4)	0 (0) – 1 (0.53)	20 (9.5) – 19 (10.1)
Cephalosporins (Cefixime, ceftriaxone)	42 (19.5) – 43 (20.9)	3 (1.46) – 3 (1.60)	18 (8.5) – 14 (7.4)
Fluroquinolones (Levofloxacin and Moxifloxacin)	34 (16.5) – 38 (18.5)	2 (0.9) – 2 (1.0)	22 (10.7) – 21 (11.2)
Macrolides (Azithromycin and Clarithromycin)	89 (43.5) – 62 (30.2)	2 (1.0) – 5 (2.67)	30 (14.6) – 18 (9.7)

**Simple T-test (interventional group only): **RTIs=respiratory tract infection: LRTs=lower respiratory tract infection*
