# Supplementary material for: Adherence to Antibacterial Therapy and Associated Factors in Lower Respiratory Infections in War-Affected Areas: A Randomized Controlled Trial
Source: Antibiotics (Basel). 2025 Sep 27;14(10):977. doi: 10.3390/antibiotics14100977 (PMC12561823; doi:10.3390/antibiotics14100977)
Supplement: Supplementary file 1 [file antibiotics-14-00977-s001.zip › 2.Supplementary-Table-S2 The general knowledge of the participants.pdf]

Supplementary-Table S2: The general knowledge of the participants

Participant's knowledge related to antibiotics and related factors antibiotic practices	Control *n* (%)	Intervention *n* (%)	*P*-value**
If someone's mucous becomes colored when having a cold, he always needs antibiotic to get rid of the cold?			0.01
Yes	134 (65.6)	114 (61.0)	
No	0 (0.0)	43 (23.0)	
Do not know	71 (34.4)	30 (16.0)	
A persistent cough (longer than one week) always needs to be treated with antibiotics to cure.			0.05
Yes	142 (69.3)	122 (65.2)	
No	0 (0.0)	35 (18.7)	
Do not know	63 (30.7)	30 (16.1)	
It is appropriate to take antibiotics when having a sore throat and a common cold with complications?			0.02
Yes	140 (68.3)	103 (55.0)	
No	8 (3.9)	36 (19.3)	
Do not know	57 (27.8)	48 (25.7)	
Chest congestion and cough in a 3–6-year-old child always need to be treated with antibiotics.			0.02
Yes	131 (63.9)	98 (53.1)	
No	26 (12.7)	53 (28.0)	
Do not know	48 (23.4)	36 (18.9)	
Respiratory infections can heal themselves without antibiotics and no need to visit a physician.			0.05
Yes	72 (35.1)	79 (41.2)	
No	92 (44.9)	78 (40.7)	
Do not know	41 (20.0)	30 (18.1)	
If one feels better after only partially completing an antibiotic course, one can terminate the therapy immediately, especially in LRTIs.			0.00
Yes	65 (31.7)	67 (35.8)	
No	75 (36.6)	81 (43.3)	
Do not know	65 (31.7)	39 (20.9)	
Do antibiotics often cause side effects such as diarrhea due to the killing of good bacteria (normal flora)?			
Yes	156 (76.1)	144 (77.0)	
No	11 (5.4)	13 (7.0)	
Do not know	38 (18.5)	30 (16.0)	

Table S2: (Continued)

Participant's knowledge related to ABs and related factors ABs practices	Control N (%)	Intervention N (%)	*P*-value**
The more antibiotics we use in society, the higher the risk that resistance develops and spreads.			0.01
Yes	103 (50.2)	106 (56.7)	
No	24 (11.7)	25 (13.4)	
Do not know	78 (38.1)	56 (29.9)	

**Pearson chi-square test (interventional group only) the independent variables tested against the education level of the participants.
