# Supplementary material for: Adherence to Antibacterial Therapy and Associated Factors in Lower Respiratory Infections in War-Affected Areas: A Randomized Controlled Trial
Source: Antibiotics (Basel). 2025 Sep 27;14(10):977. doi: 10.3390/antibiotics14100977 (PMC12561823; doi:10.3390/antibiotics14100977)
Supplement: Supplementary file 1 [file antibiotics-14-00977-s001.zip › 3.Supplementary Material Table-S3 Response towards WHO questionnaire (Likert scale) by participants.pdf]

Supplementary Material Table-S3: Response towards WHO questionnaire (Likert scale) by participants.

Questions	Response	Control *n* (%)	Intervention *n* (%)
Antibiotic resistance occurs when your body becomes resistant to ABs, and they no longer work as well.	True	95 (46.3)	154 (82.4)
	False	29 (14.2)	0 (0.0)
	Do not know	81 (39.5)	33 (17.6)
Many infections are becoming increasingly resistant to treatment by antibiotics.	True	66 (32.2)	153 (81.8)
	False	35 (17.1)	0 (0.0)
	Do not know	104 (50.7)	34 (18.2)
If bacteria are resistant to antibiotics, it can be very difficult or impossible to treat the infections they cause	True	111 (54.1)	156 (83.4)
	False	33 (16.1)	0 (0.0)
	Do not know	61 (29.8)	31 (16.6)
Antibiotic resistance is an issue that could affect me or my family	True	65 (31.7)	148 (79.1)
	False	29 (14.1)	0 (0.0)
	Do not know	111 (54.2)	39 (20.9)
Antibiotic resistance is an issue in other countries but not here	True	69 (33.6)	0 (0.0)
	False	42 (20.5)	142 (75.9)
	Do not know	94 (45.9)	45 (24.1)
Antibiotic resistance is only a problem for people who take antibiotics regularly	True	94 (45.9)	134 (71.7)
	False	99 (48.2)	26 (13.9)
	Do not know	12 (5.9)	27 (14.4)
Bacteria that are resistant to antibiotics can be spread from person to person	True	88 (42.9)	113 (60.4)
	False	117 (57.1)	74 (39.6)
	Do not know	0 (0.0)	0 (0.0)
Antibiotic-resistant infections could make medical procedures like surgery, organ transplants, and cancer treatment much more dangerous	True	70 (34.1)	113 (60.4)
	False	67 (32.7)	29 (15.5)
	Do not know	68 (33.2)	45 (24.1)
