# Supplementary material for: Adherence to Antibacterial Therapy and Associated Factors in Lower Respiratory Infections in War-Affected Areas: A Randomized Controlled Trial
Source: Antibiotics (Basel). 2025 Sep 27;14(10):977. doi: 10.3390/antibiotics14100977 (PMC12561823; doi:10.3390/antibiotics14100977)
Supplement: Supplementary file 1 [file antibiotics-14-00977-s001.zip › 4.Supplementary Material Table S4. Trial participants (intervention and control) responses on antibiotic resistance.pdf]

Supplementary Material Table S4: Trial participants (intervention and control) responses on antibiotic resistance.

Participant knowledge related to antibiotic resistance	Control<br>*n* (%)	Intervention<br>*n* (%)	*P*-value**
Antibiotic resistance happens when germs like bacteria develop the ability to defeat the drugs designed to kill them.			0.05
Yes	97 (47.3)	88 (47.1)	
No	21 (10.2)	21 (11.2)	
Do not know	87 (42.5)	78 (41.7)	
Nowadays, antibiotic resistance is a big problem in the world, particularly in Pakistan.			0.02
Yes	64 (31.2)	103 (55.2)	
No	0 (0.0)	24 (12.8)	
Do not know	141 (68.8)	60 (32.0)	
ABR can develop because of improper usage of antibiotics.			0.00
Yes	66 (32.2)	76 (40.6)	
No	42 (20.5)	40 (21.4)	
Do not know	91 (47.3)	71 (38.0)	
Doctors always undertake a comprehensive examination to determine whether a patient requires antibiotics and talk about antibiotic resistance *(Physician role)*			0.19
Yes	81 (39.5)	94 (50.3)	
No	124 (60.5)	93 (49.7)	
Do not know	0 (0.0)	0 (0.0)	
When antibiotics are prescribed for RTIs, the doctor takes time to provide information on how they should be used, in an understandable manner. *(Physician role)*			0.31
Yes	18 (8.8)	34 (18.2)	
No	174 (84.9)	153 (81.8)	
Do not know	13 (6.3)	0 (0.0)	
I usually know whether I need antibiotics before I go to the doctor, and I trust a doctor's decision if he or she does not. *(Misuse of antibiotics)*			0.00
Yes	99 (48.3)	103 (55.1)	
No	37 (18.0)	39 (20.8)	
Do not know	69 (33.7)	46 (24.1)	
The pharmacy staff takes the time to explain how to utilize medications properly *(Pharmacist role)*			0.01
Yes	72 (35.1)	69 (36.9)	
No	97 (47.3)	97 (51.9)	
Do not know	26 (17.6)	21 (11.2)	
The pharmacist was present at the time of antibiotic dispensing?			0.70

Yes	18 (8.8)	24 (12.8)	
No	187 (91.2)	137 (73.3)	
Do not know	0 (0.0)	26 (13.9)	
Pharmacist only provides ABs only on prescription.			0.01
Yes	130 (63.4)	119 (63.6)	
No	63 (30.7)	18 (9.6)	
Do not know	12 (5.9)	50 (26.8)	

**Pearson chi-square test (interventional group only) the independent variables tested against the education level of the participants.

Overall adherence comparison.

MMAS-8 categories	Control *n* (%)	Intervention *n* (%)
Low adherence	125 (61.2)	32 (17.1)
Medium adherence	66 (32.0)	60 (32.1)
Higher adherence	14 (6.8)	95 (50.8)
