# Supplementary material for: Adherence to Antibacterial Therapy and Associated Factors in Lower Respiratory Infections in War-Affected Areas: A Randomized Controlled Trial
Source: Antibiotics (Basel). 2025 Sep 27;14(10):977. doi: 10.3390/antibiotics14100977 (PMC12561823; doi:10.3390/antibiotics14100977)
Supplement: Supplementary file 1 [file antibiotics-14-00977-s001.zip › 5.Supplementary Material Table S5. Comparison of MMAS-8 scores with participant demographics.pdf]

Supplementary Material Table S5: Comparison of MMAS-8 scores with participant demographics.

Variables	Control (*n*)	Intervention (*n*)	*P*-value
Gender			
Male	187	176	0.27
Female	18	11	
Age (years)			
18-23	58	59	0.30
24-29	50	47	
30-35	37	41	
36-41	37	29	
>41	23	11	
Monthly income (PKR)			
>10000-<20000	70	61	0.10
>20000-<300000	68	69	
>30000-<40000	34	39	
>40000-<50000	2	4	
>50000	31	14	
Past medical history			
Yes	60	54	0.90
No	145	133	
Education			
Illiterate	33	26	0.07
Primary/Secondary	84	56	
Intermediate	70	74	
Bachelors or higher	18	31	
MMAS-8 items QA scores	Mean ± Standard Deviation	Mean ± Standard Deviation	*P*-value
Mean score	6.4 ± 2.1	7.8 ± 2.1	≤0.001
