# Supplementary material for: Adherence to Antibacterial Therapy and Associated Factors in Lower Respiratory Infections in War-Affected Areas: A Randomized Controlled Trial
Source: Antibiotics (Basel). 2025 Sep 27;14(10):977. doi: 10.3390/antibiotics14100977 (PMC12561823; doi:10.3390/antibiotics14100977)
Supplement: Supplementary file 1 [file antibiotics-14-00977-s001.zip › 7.Supplementary Material Table S7 Def.pdf]

Supplementary Material Table S7: Details of a text message description of the contents

Category	Details of the message
Knowledge related to LRTIs	LRTIs are infections that occur in the lower respiratory tract and cause infection which leads to COPD, ACOPD, bronchitis, and others. Such infections can further appear with Pneumonia and sometimes cannot be treated with antibiotics.
Knowledge related to ABs	Antibiotics are different from other medications and if you miss your dose on time, it can affect your therapeutic outcomes.
Modification in daily routine	Set an alarm for dose time, write the dose timing on separate paper, or tell someone to remind your antibiotic doses on time.
Medication adherence and improvement	Please remember to take your antibiotics as directed (> 5/7) each day. To help you remember to take your meds every day, place them on your table or drinking vessel. Please monitor your prolonged cough from a chest infection and seek medical attention if you notice any anomalies.
