# Supplementary material for: Adherence to Antibacterial Therapy and Associated Factors in Lower Respiratory Infections in War-Affected Areas: A Randomized Controlled Trial
Source: Antibiotics (Basel). 2025 Sep 27;14(10):977. doi: 10.3390/antibiotics14100977 (PMC12561823; doi:10.3390/antibiotics14100977)
Supplement: Supplementary file 1 [file antibiotics-14-00977-s001.zip › 8.Supplementary Material Table S8.1&8.2-SPIRIT and Blinding.pdf]

Supplementary Material Table S8.1: SPIRIT-based Enrollment, intervention, and evaluation schedules

	STUDY TIME								
	Enrollment	Allocation	Post allocation						Time (close-out)
TIME POINT	Time-1	W-1	W-2	W-3	W-4	W-5	W-6	W-7	
ENROLLMENT:									
Patient eligibility	x								
Consent	x								
Baseline data	x								
Patient allocations									
Interventions:									
Leaflets			←	—	—	—	—	—	→
Booklets (CG)									
awareness (WHO)			←	—	—	—	—	—	→
Usual care									
ASSESSMENT:									Outcome's analysis
Baseline characteristics	x	x	X	x	X	X	x	X	
Therapy success rate	x	x	X	x	X	X	x	X	
Primary outcomes									
Overall adherence	x	x	X	x	X	X	x	X	
Secondary Outcomes									

**W=week*

Supplementary Material Table S8.2: Blinding approach for the entire Study

Stakeholders	Allocation	Interventions	CPhs	Assessment	Analysis
Observer of the trial (P-I)	(a)	(a)	(a)	(a)	(a)
Trial-participants	n-a	n-a	n-a	n-a	n-a
Duty-pharmacists	n-a	(a)	(a)	n-a	n-a
DCTs	n-a	n-a	n-a	n-a	n-a
Data-analysts	n-a	n-a	n-a	n-a	(a)

Aware: a; not-aware: n-a
