# Supplementary material for: Adherence to Antibacterial Therapy and Associated Factors in Lower Respiratory Infections in War-Affected Areas: A Randomized Controlled Trial
Source: Antibiotics (Basel). 2025 Sep 27;14(10):977. doi: 10.3390/antibiotics14100977 (PMC12561823; doi:10.3390/antibiotics14100977)
Supplement: Supplementary file 1 [file antibiotics-14-00977-s001.zip › 9.Supplementary -Table S9- Mean differences and overall scores..pdf]

Supplementary -Table S9: Mean differences and overall scores.

Overall Score	Control Mean (SD±)	Intervention Mean (SD±)	Mean Difference
PAS-WHO	1.6 (0.69)	2.1 (0.63)	0.5
BMQ	1.3 (0.5)	2.4 (0.60)	1.1
