# Supplementary material for: Adherence to Antibacterial Therapy and Associated Factors in Lower Respiratory Infections in War-Affected Areas: A Randomized Controlled Trial
Source: Antibiotics (Basel). 2025 Sep 27;14(10):977. doi: 10.3390/antibiotics14100977 (PMC12561823; doi:10.3390/antibiotics14100977)

Supplementary Material Figure-S1: Different sources of information based on full, partial, and no information obtained by the participants and each question as top legend of individual Figure (a-e).

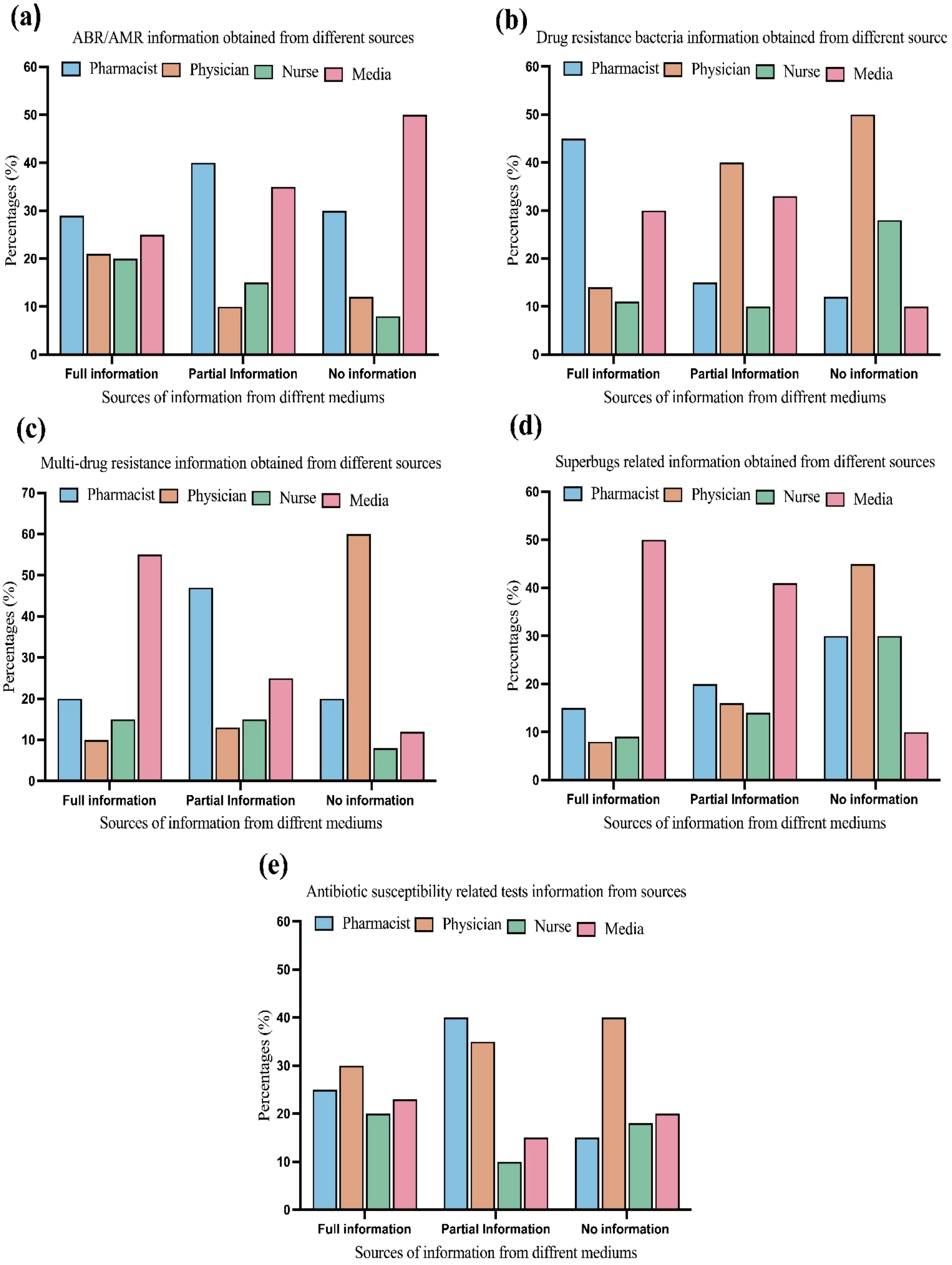

Supplement: Supplementary file 1 [file antibiotics-14-00977-s001.zip › F1.Supplementary Material Figure-S1.pdf]
