# Supplementary material for: Adherence to Antibacterial Therapy and Associated Factors in Lower Respiratory Infections in War-Affected Areas: A Randomized Controlled Trial
Source: Antibiotics (Basel). 2025 Sep 27;14(10):977. doi: 10.3390/antibiotics14100977 (PMC12561823; doi:10.3390/antibiotics14100977)
Supplement: Supplementary file 1 [file antibiotics-14-00977-s001.zip › F2.Supplementary Material Figure-S2. Brochure for the participants based on educational intervention.pdf]

Supplementary Material Figure S2: Brochure for the participants based on educational intervention

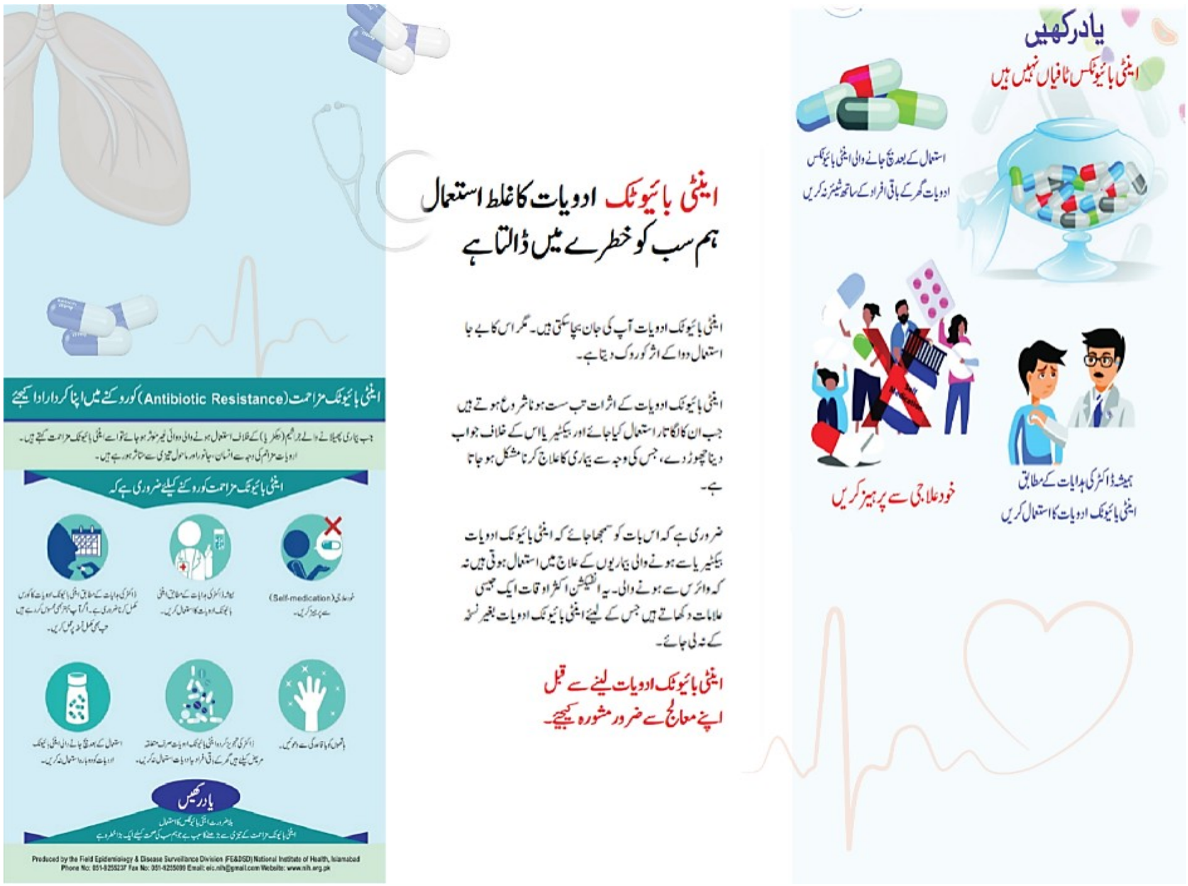

اینٹی بائیوٹکس لیتے وقت احتیاط برتیں

آپ کو ذاتی حفظان صحت کو فروغ دینا چاہیے تاکہ آپ اور آپکے گھرانے کی صحت کی حفاظت ہو پائے۔
نسخہ کے بغیر اینٹی بائیوٹکس کبھی نہ لیں۔
اینٹی بایوٹکس لینے کے حوالے سے اپنے ڈاکٹر کے مشورے پر عمل کریں۔
اپنی تجویز کردہ اینٹی بائیوٹکس کو پانی یا دودھ کے ساتھ کھانے سے پہلے اور بعد میں لے سکتے ہیں لیکن فارمیسی میں ہماری ٹیم کی طرف سے دی گئی ہدایات پر عمل کریں۔
اپنی تجویز کردہ اینٹی بایوٹک کو صحیح وقت پر لیں اور اینٹی بائیوٹک کی خوراک کے وقت کا خیال رکھیں۔
مثال کے طور پر، آپ نے پہلی خوراک صبح 9 بجے لی ہے لہذا اگلی خوراک صبح 9 بجے ہی لینی چاہیے اور اگر دو خوراکیں ہیں تو صحیح وقت کے وقفے کو یقینی بنائیں۔
کم از کم پانچ دن یا زیادہ سے زیادہ سات دن تک اپنی اینٹی بائیوٹکس لیں۔
فارمیسی میں ہماری ٹیم کی ہدایات کے مطابق عمل کریں۔
تمام تجویز کردہ اینٹی بائیوٹک خوراکیں ہدایات کے مطابق لیں اور اس بات کو یقینی بنائیں کہ کوئی بھی خوراک ضائع نہ ہو۔
معیاد ختم ہونے والی اینٹی بایوٹک کو گھر میں نہ رکھیں اور نہ ہی اسے خاندان کے دیگر افراد کے ساتھ بانٹیں۔
گھر میں غیر ضروری اینٹی بائیوٹک کو ذخیرہ نہ کریں۔
بچوں کو اینٹی بائیوٹک کی پہنچ سے دور رکھیں۔
اینٹی بائیوٹکس لیتے ہوئے اپنے ڈاکٹر کے مشورے پر عمل پیرا ہوں اور خود سے اینٹی بائیوٹکس لینا ترک نہ کریں حتی کہ آپ بہتر محسوس کر رہے ہوں۔
اگر کوئی شک ہو تو اپنے ڈاکٹر یا فارمسسٹ سے مشاورت کریں۔
نسخہ کے بغیر اینٹی بائیوٹکس کبھی نہ لیں۔
اگر آپ اوپر دی گئی ہدایات پر عمل نہیں کرتے ہیں، تو آپ کی اینٹی بائیوٹکس آپ کے علاج میں بیکار ہوسکتی ہیں۔
اور اینٹی بائیوٹکس کے خلاف بیکٹیریا کی مزاحمت کے امکانات بڑھ جائیں گے۔

(AMR) اینٹی بائیوٹک/مائکروبیل ریزسٹنس

(AMR)اینٹی بائیوٹک/مائکروبیل ریزسٹنس کیا ہے؟
اینٹی بائیوٹک/مائیکروبیل مزاحمت اس وقت ہوتی ہے جب جراثیم جیسے بیکٹیریا سابقہ موثر ادویات (جیسے جراثیم) کے خلاف مزاحمت ظاہر کرتے ہیں۔

اے ایم آر کے نتائج کیا ہیں؟
اگر اے ایم آر کا مسئلہ بہتر نہیں ہوتا تو، موجودہ اینٹی مائیکروبیلز غیر موثر ہو جائیں گی اور انفیکشنز قابل علاج نہ رہیں گے، جس کا نتیجہ طویل بیماری اور موت کے بڑھتے ہوئے خطرے کی صورت میں نکلتا ہے۔

عامۃ الناس کو اے ایم آر کا مقابلہ کرنے کے لیے کیا کرنا چاہیے؟
اے ایم آر ہر ایک کو متاثر کر رہی ہے۔ عامۃ الناس کے ارکان کو درج ذیل پر عمل پیرا ہو کر اے ایم آر سے مقابلہ آرائی کے حوالے سے کلیدی کردار بھی ادا کرنا چاہیے۔

Name of Antibiotics
(Fill by Pharmacist)

1.______________ 2.______________ 3.______________
4.______________ 5.______________

Dose Strength:

200mg_____ 400mg_____ 250mg_____ 500mg_____

اینٹی بائیوٹک خوراک کا وقفہ

دن	پہلی خوراک	دوسری خوراک	تیسری خوراک	چوتھی خوراک
پیر				
منگل				
بدھ				
جمعرات				
جمعہ				
ہفتہ				
اتوار				

اینٹی بائیوٹکس کا موزوں استعمال

- اپنے ڈاکٹر سے اینٹی بائیوٹکس طلب نہ کریں
- اینٹی بائیوٹکس لیتے ہوئے اپنے ڈاکٹر کے مشورہ پر عمل کریں
- چاہے آپ بہتر محسوس کر رہے ہوں مگر آپ اینٹی بائیوٹکس خود سے لینا بند نہ کریں
- بچ رہنے والی اینٹی بائیوٹکس نہ لیں
- اپنی اینٹی بائیوٹکس کی دیگر کے ساتھ شراکت نہ کریں
- نسخے کے بغیر اینٹی بائیوٹکس خود سے نہ خریدیں
- یقینی بنائیں کہ آپ کی ویکسینیشن اپ ڈیٹ شدہ ہو
- کھانسی کے آداب کا خیال رکھیں، اگر تنفسی مسائل ہوں تو سرجیکل ماسک پہنیں

Lower Respiratory Tract Infections (LRTIs)

Lower Respiratory Tract Infections(LRTIs)
کے مریضوں میں اینٹی بائیوٹکس کی پابندی کو بڑھانے کے لیے فارماسسٹ کی رہنمائی

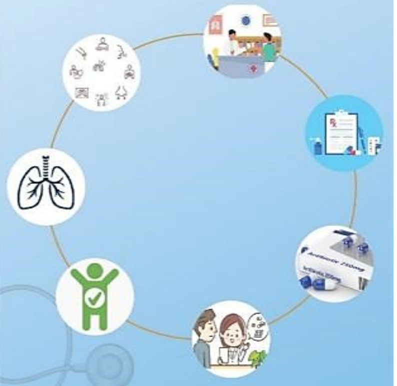

Pharmacist led educational intervention to enhance patient adherence to antibiotics for patients with lower respiratory tract infections (LRTIs)
