# Supplementary material for: Adherence to Antibacterial Therapy and Associated Factors in Lower Respiratory Infections in War-Affected Areas: A Randomized Controlled Trial
Source: Antibiotics (Basel). 2025 Sep 27;14(10):977. doi: 10.3390/antibiotics14100977 (PMC12561823; doi:10.3390/antibiotics14100977)
Supplement: Supplementary file 1 [file antibiotics-14-00977-s001.zip › Supplementary -Table S6.1 and S6.2.pdf]

Supplementary -Table S6.1: The WHO-PAS Likert Scale responses and Demographics association.

Demographics	Control Group		Intervention Group	
	Mean Rank	*P-Value	Mean Rank	*P-Value
**Gender**		0.02		0.03
Male	100.8		93.1	
Female	132.9		107.6	
Education		0.11		0.09
No Formal Education	57.9		35.9	
Formal (primary/secondary)	59.4		44.0	
Intermediate	46.4		54.5	
Bachelor or >	36.7		49.1	
**Age (years)**		0.20		0.71
18-23	58.0		55.3	
24-29	55.8		51.2	
**30-35**	33.2		46.4	
**36-41**	25.4		33.7	
**>41**	30.2		21.3	
**Monthly Income**				0.07
>10000 < 20000	69.2	0.77	60.0	
> 20000 < 300000	68.7		70.3	
>30000 < 40000	21.2		21.6	
> 40000 < 500000	17.0		25.1	
> 500000	15.1		9.8	
**Past Medical History**		0.06		0.08
Yes	106.1		92.8	
No	101.2		94.4	

**Mann–Whitney U test.*

Supplementary -Table S6.2: The BMQ responses and Demographics association based on the Mann–Whitney U test.

Demographics	Control Group		Intervention Group	
	Mean Rank	*P-Value	Mean Rank	*P-Value
**Gender**		0.14		0.82
Male	101.6		93.8	
Female	121.4		97.1	
Education		0.03		0.82
No Formal Education	50.8		35.6	
Formal (primary/secondary)	62.2		44.2	
Intermediate	43.8		54.0	
Bachelor or >	46.7		50.4	
**Age (years)**		0.81		0.12
18-23	52.2		50.9	
24-29	62.1		56.6	
**30-35**	34.2		34.1	
**36-41**	35.8		37.4	
**>41**	27.2		16.3	
**Monthly Income**		0.37		0.25
>10000 < 20000	66.6		62.0	
> 20000 < 300000	71.6		68.5	
>30000 < 40000	21.3		21.6	
> 40000 < 500000	11.0		25.1	
> 500000	15.3		8.8	
**Past Medical History**		0.57		0.54
Yes	90.4		90.6	
No	95.1		95.3	

*Mann–Whitney U test.
